# Integrated Phenotypic and Genomic Characterization of Endophytic *Bacillus vallismortis* E4 for Biopolymer and Lipopeptide Production

**DOI:** 10.3390/life16101707

**Published:** 2026-10-12

**Authors:** Maria Muhammad Ashraf, Khwaja Ali Hasan, Shahida Naz, Mohammed Alorabi, Amal S. Alswat, Muhammad Sohail

**Affiliations:** 1 Department of Microbiology, University of Karachi, Karachi 75270, Pakistan; 2 Molecular and Structural Biochemistry Research Laboratory, Department of Biochemistry, University of Karachi, Karachi 75270, Pakistan; 3 Department of Biotechnology, College of Sciences, Taif University, Taif 21944, Saudi Arabia

**Keywords:** biopolymer, biosurfactant, exopolysaccharide, genomic characterization, lipopeptide, polyhydroxybutyrate

## Abstract

Biopolymers are valuable alternatives to plastics because of their biocompatibility and biodegradability. Biopolymers and lipopeptides have been documented for *Bacillus vallismortis*; however, its whole-genome sequence (WGS) remains understudied and scarce in public databases. This study analyzed the WGS of endophytic *B. vallismortis* E4 and its potential for biopolymer and lipopeptide production. The genome showed 43.83% GC content with 4,128,106 bp in a total of 76 contigs. Functional annotation revealed 4167 genes, with 3943 protein-coding sequences. The genome contained genes for nitrogen fixation, stress tolerance, and metabolic pathways relevant to plant growth-promoting attributes. Genes for biosurfactant and polysaccharide synthesis were identified. PHB-related genes were limited to acetyl-CoA acetyltransferase and 3-hydroxybutyryl-CoA dehydrogenase, and canonical *phaC* and *phaB* were not detected. Nonetheless, these detected genes were complemented by the corresponding phenotypes. Biosurfactant production was confirmed by oil displacement area (36.65 ± 0.38 cm^2^) and emulsification index (49.66 ± 3.51%). PHB production was low (5.47 ± 0.12% of dry cell weight) and requires optimization. FTIR preliminarily identified the polymer as PHB, and SEM showed a slightly rough surface with spherical structures. The yield of cell-associated EPSs was 35.3 ± 0.1 mg/100 mL and was visualized using FTIR spectra, while AFM and XRD indicated its spherical and crystalline structure, respectively. The preliminary non-pathogenicity assessment of E4 suggests its suitability for industrial use, although the presence of diverse AMR genes needs to be further validated through physiological and phenotypic studies.

## 1. Introduction

Microbial cell factories (MCFs) are sources of bioproducts, including nutrients, biochemicals, and pharmaceuticals. These are considered to be chips that can support the emerging era of the bioeconomy [1]. These bioproducts are of great interest for industrial processes owing to their biocompatibility, eco-friendliness, and biodegradability [2], which augments their demand in food packaging, electronics, pharmaceuticals, drug delivery, cosmetics, construction, and nutraceuticals [3,4]. Their global market share is expected to reach $50 billion by 2030, with a compound annual growth rate (CAGR) of 7.9%-15.6% [1]. In this respect, MCFs have gained considerable attention because of their short production time, ease of handling, and bulk production capabilities. Microorganisms produce different biomolecules, including exopolysaccharides (EPSs) (such as curdlan, dextran, and hyaluronic acid), polyglutamic acid, biosurfactants, and biopolyesters in the form of polyhydroxybutyrate (PHB) granules [5,6,7,8,9].

The biodegradability and eco-friendliness of PHB make it a sustainable alternative to conventional petrochemical-based polymers. Globally, the current annual plastic production exceeds 460 million tons, of which approximately 9% is recycled [10]. This massive production and improper disposal and landfilling have raised concerns regarding health and environmental issues [11]. PHB biopolyesters are accumulated by bacteria in the form of granules, which serve as reserves for their survival and succession in microenvironments [8]. Hence, biological alternatives offer environmentally friendly and sustainable solutions to these problems.

Bacterial exopolysaccharides (EPSs) are high-molecular-weight carbohydrate polymers that are either released into the medium (free or ‘slime’ EPSs) or remain attached to the cell surface as a capsule (capsular or cell-associated polysaccharides) [12]. They exhibit antitumor, antioxidant, antiviral, and immunostimulatory activities [12]. These polysaccharides act as hydrocolloids to modulate interfacial behavior, viscosity, and gelation properties [13]. These hydrocolloids are used as food additives to improve quality and flavor as gelling, emulsifying, thickening, and stabilizing agents. Their applications further extend to pharmaceuticals and cosmetics [14]. In agriculture, EPSs aid in the cohesion, adhesion, water retention, or aggregation of soil particles and act as a potential barrier to protect bacteria from the accumulation of toxic compounds in the rhizospheric region [15].

Biosurfactants, which are surface-active amphiphilic molecules, enhance the solubility of two immiscible fluids by reducing their surface tension, resulting in the emulsification of the two phases [16]. They have a low molecular weight, stabilize emulsions, and are similar to lipopeptides, glycolipids, heteropolysaccharides, or lipopolysaccharides [17]. They are extensively used as therapeutic agents for wound healing and against multidrug-resistant microbial infections owing to their antimicrobial and antiviral properties [18]. Biosurfactants induce systemic resistance in plants owing to their antimicrobial properties, enhance nutrient uptake, and aid in colonization by plant growth-promoting bacteria (PGPB). Moreover, they promote root development, leading to improved plant growth [19,20].

Endophytes are microorganisms that reside at different sites in plant tissues without causing any harm. PGPBs, when isolated from halophytes or saline soil, are of particular interest because they produce versatile metabolites that enable survival under harsh conditions [21]. Endophytes are also known for their production of different macromolecules, mostly reported in the genus *Bacillus* [8,9].

*B. vallismortis* is closely related to *B. subtilis*, as they are phenotypically similar, but they vary in their whole-cell fatty acid composition. *B. vallismortis* is a biofilm producer, motile, spore-forming, Gram-positive, rod-shaped bacterium with blunt ends (ellipsoidal to cylindrical that can be located subterminally, centrally, or paracentrally) [15,22,23]. According to De Vos et al. [23], *B. subtilis* and related species grow optimally at 28–30 °C and pH 5.5–8.5. They normally grow in the presence of 7% (*w*/*v*) NaCl but can survive up to 10% NaCl. This feature makes them ideal for saline agriculture and other biotechnology applications.

*B. vallismortis* has demonstrated tremendous potential for mitigating plant stress through ACC deaminase production, making nutrients available to plants, and producing plant growth hormones, including indole acetic acid, gibberellic acid, and cytokinin. It is a sustainable source of industrial enzymes, such as amylase, pectinase, chitinase, laccase, endoglucanase, and lignin peroxidase [15]. Different studies have reported it as an endophyte and its role as a PGPB in tobacco, apple, canola, kale, rice, tea, and tomato plants through phosphorus acquisition, inhibiting fungal and bacterial phytopathogens, and stimulating plant defense through biofilm and volatile organic compound production [15,22,24,25,26,27,28,29]. As *B. vallismortis* is a promising source of biosurfactants, cell-associated EPS, and PHB, we previously examined the enzymatic potential of strain E4 [30]. Although different studies have been conducted on *B. vallismortis*, its whole-genome sequence (WGS) has not yet been reported in Pakistan.

This study focused on the in silico analysis of WGS to investigate its stress-alleviating potential at the genomic level. Furthermore, genomic characterization was focused on less-explored traits, including biosurfactant, PHB, and EPS production, considering their applicability in different sectors. The genetic characterization of biopolymers and lipopeptides was confirmed experimentally through their production and subsequent analysis. Biosurfactant production was examined using an emulsification index, whereas PHB was validated using Fourier transform infrared spectroscopy (FTIR) and scanning electron microscopy (SEM). EPS was characterized using FTIR, atomic force microscopy (AFM), and X-ray diffraction (XRD). Genomic analysis of *B. vallismortis* E4 revealed its remarkable genetic potential to promote plant growth and survival under extreme conditions. This will pave the way for its use as a biocontrol agent or as a PGPB to facilitate the agricultural sector in future research.

## 2. Materials and Methods

### 2.1. Source of Strain, Collection and Identification

The endophytic *B. vallismortis* strain E4 was isolated from the roots of *A. macrostachyum*, collected from Sandspit, Karachi, Pakistan (24°50′24.4″ N, 66°54′40.3″ E) in February 2020. This strain has been reported to produce various enzymes, such as amylase, xylanase, pectinase, cellulase, and lipase. This strain was initially identified based on the partial sequence of 16S rDNA and reported as *Bacillus* sp. (GenBank accession number ON060356.1) [30].

### 2.2. Extraction of Genomic DNA

To extract genomic DNA using the cetyltrimethylammonium bromide (CTAB) method, the culture was grown on tryptone soy agar (TSA; Oxoid, Hampshire, UK) at 30 °C for 18 h, and an axenic culture was obtained. A single isolated colony from TSA was transferred to tryptone soy broth (Oxoid, UK) and incubated at 30 °C for 24 h. The cells were separated by centrifugation at 5000× *g* for 20 min, and the cell pellet was used for whole-genome extraction as described previously [31]. The extracted DNA was further evaluated for purity and quality using a NanoDrop 2000 spectrophotometer (Thermo Fisher Scientific, Waltham, MA, USA) by measuring the absorbance ratios (A260/280 and A260/230). The integrity of the extracted DNA was assessed using 1% (*w*/*v*) agarose gel electrophoresis.

### 2.3. Genome Sequencing and Assembly

Paired-end reads (2 × 150 bp) were generated using the DNBSEQ-G400 platform (MGI Tech Co., Ltd., Shenzhen, China). Read quality was assessed using FastQC (v0.12.1). Sequences were subsequently quality-filtered using the commands (https://usegalaxy.org.au/; accessed on 24 July 2024) and assembled into contigs using SPAdes (https://usegalaxy.org.au/; accessed on 24 July 2024) [32]. A draft genome assembly was assessed for quality, completeness, contamination, and coverage depth using QUAST (v5.0.2) and CheckM2 (v1.1.0) analyses using default parameters on the Galaxy server (https://usegalaxy.org.au/; accessed on 24 July 2024) [33,34]. QUAST works on the analysis of contigs and the alignment of contigs to( the reference using minimap2. All tools were run with default parameters unless otherwise stated. The whole-genome data of *B. vallismortis* E4 were submitted to GenBank under accession number JBIMFA000000000.

### 2.4. Phylogenetic Analysis

Phylogenetic analysis was performed based on digital DNA–DNA hybridization (dDDH), multilocus sequence typing (MLST) of the core genome, and ribosomal multi-locus sequence typing (rMLST). The Type (Strain) Genome Server (TYGS) (https://tygs.dsmz.de/; accessed on 4 February 2026) was used to construct a phylogenetic tree using the whole-genome attributes [35]. dDDH values were calculated using the Genome-to-Genome Distance Calculator (GGDC) via TYGS and are reported for formula d4, which is independent of genome length, recommended for draft genomes, and is the formula to which the 70% species threshold applies [35]. The average nucleotide identity was determined using autoMLST 2.0 [36].

### 2.5. Genome Annotation

The genome was annotated using the NCBI Prokaryotic Genome Annotation Pipeline (PGAP). The Rapid Annotation and Subsystem Analysis Tool Kit (RASTtk) pipeline (https://rast.nmpdr.org/rast.cgi; accessed on 12 September 2025) and PATRIC were used for functional annotation of the genome [37,38]. A genomic map was constructed using Proksee (https://proksee.ca/; accessed on 12 September 2025) [39].

### 2.6. Genes Related to Endophytic Behavior, Biopolymer and Lipopeptide Production

Genes related to endophytic behavior, including colonization, nutrient exchange, and stress mitigation (salt tolerance), including those for transporter proteins, nitrogenase (nitrogen fixation), sulfur metabolism, endoglucanases (cell wall degradation), lipases, and phytohormone production, were annotated using the Rapid Annotation and Subsystem Analysis tool kit (RASTtk) (https://rast.nmpdr.org/rast.cgi; accessed on 12 September 2025) and the PATRIC database. Furthermore, genes conferring the ability to produce biopolymers, such as PHB, exopolysaccharides (EPS), and lipopeptides (biosurfactants), were also detected using RASTtk. In addition, secondary metabolite-controlling genes were evaluated using AntiSMASH (bacterial version) [40].

### 2.7. Genome-Based Safety Assessment (GBSA)

Genes were analyzed for homology with databases of virulence factors, antibiotic resistance, and drug targets to assess whether the strain posed a hazard using the ABRicate (v1.0.1) and PathogenFinder 2 (v0.6.0) tools [41].

### 2.8. Detection of Biopolymers and Lipopeptide Production

#### 2.8.1. Production Media and Inoculum Preparation

A single colony of *B. vallismortis* E4 from a nutrient agar plate was inoculated into 50 mL of nutrient broth and incubated at 30 °C and 120 rpm for 24 h. Cells were harvested by centrifugation (6000 rpm for 10 min), washed once, and resuspended in 5 mL sterile 0.85% NaCl. This suspension was added to the production medium at a volume required to obtain an initial OD_600_ of 0.3. The composition of MSM (g/L) was as follows: yeast extract (10), K_2_HPO_4_ (2), KH_2_PO4 (1), MgSO_4_∙7H_2_O (0.5), KCl (0.1), FeSO_4_∙7H_2_O (0.02), CaCl_2_ (0.01), glycerol (10), and ground *Cassia fistula* seeds (5). The MSM used for EPS production had the following composition (g/L): NH_4_NO_3_ (0.25), K_2_HPO_4_ (1.5), KH_2_PO_4_ (2), CaCl_2_ (1), Na_2_SO_4_ (1), and starch (10).

#### 2.8.2. Preparation of *Cassia fistula* (CF) Seeds for the Production of Biosurfactants and PHB

The seeds of *C. fistula* were harvested from *C. fistula* plants on the premises of the University of Karachi, Pakistan. The seeds were dried, ground using a coffee bean grinder (Anex Germany Products GmbH, Kirchheim-Teck, Germany), and sieved through a 300 µm mesh. The ground CF seed powder was stored in an airtight container until further use. The ground seeds were used as a crude carbon source in MSM for biosurfactant and PHB production.

#### 2.8.3. Lipopeptide (Biosurfactant) Production

The biosurfactants produced by *B. vallismortis* E4 were evaluated in MSM using a specific agro-industrial waste, CF ground seeds. Briefly, MSM inoculated with fresh culture was incubated at 30 °C with an agitation rate of 120 rpm. After 48 h, the supernatant was collected by centrifugation at 4500 rpm and analyzed for the presence of biosurfactants. The supernatant was acidified with 1M HCl until a pH of 2.0 was achieved. The acidified suspension was incubated for 24 h at 4 °C and centrifuged to collect the precipitates of acidic anionic biosurfactants [42]. The presence of biosurfactant was analyzed using the oil spread test, measuring oil displacement area, and emulsification assay. In the following tests, sodium dodecyl sulfate was used as a positive control as a surfactant, while phosphate-buffered saline (PBS) and supernatant of the uninoculated MSM were used as negative controls.

##### Oil Spread Test

Burned diesel oil (20 µL) was carefully added to the surface of distilled water (40 mL) in a Petri dish, followed by the addition of the crude supernatant (10 µL). The appearance of a clear zone indicated the presence of biosurfactants in the supernatant [43].

##### Oil Displacement Area

The displacement area of the oil was determined by adding the supernatant (0.5 mL) to the burned diesel oil (1 mL) that was placed on the surface of distilled water (40 mL). The radius of the clear zone was measured. The area of displacement was calculated using the following formula [44]:

Oil displacement area (cm^2^) = 3.14 × r^2^

where ‘r’ indicates the radius of the clear zone.

##### Emulsification Assay

The emulsification ability of the biosurfactant was tested by adding the supernatant in equal volume to olive oil, homogenizing, and leaving for 30 min. The height of the emulsion was recorded, and the emulsification index was calculated using the following formula [45]:

Emulsification index % = (height of emulsion/height of total solution) × 100

#### 2.8.4. PHB Production

The ability to produce PHB under shake-flask conditions was investigated in MSM supplemented with 1% glucose instead of glycerol. Freshly prepared inoculum was transferred as described in Section 2.8.1 of *B. vallismortis* E4, transferred to MSM, and incubated at 30 °C with agitation (120 rpm) for 48 h.

##### Extraction of PHB

After incubation, the medium was centrifuged at 4500 rpm for 20 min to remove the cells. The supernatant was discarded, and the cells were washed with phosphate-buffered saline and dried to measure the dry cell weight. The cells were incubated with 5 mL of 4% (*v*/*v*) hypochlorite and 5 mL of chloroform at 37 °C for 90 min at 120 rpm to extract PHB. The suspension was centrifuged at 4500 rpm for 25 min to obtain the precipitate. The sodium hypochlorite-containing cell debris was discarded, and the chloroform phase, which contained PHB, was collected using filter paper. PHB was recovered by evaporating chloroform [46].

##### Characterization of PHB

The surface morphology of the PHB was analyzed using scanning electron microscopy (SEM). The dried PHB sample was coated with a thin layer of gold using an ion-sputtering device (JEC-1500, JEOL Ltd., Akishima, Tokyo, Japan). The samples were analyzed at 20 kV using SEM (JEOL Ltd., Akishima, Tokyo, Japan). The corresponding functional groups in PHB were detected using an IR Prestige-21 spectrometer (Shimadzu Corporation, Kyoto, Japan) with a KBr-PHB fused disk. The spectra were analyzed using the LabSolutions IR 2.13 software.

#### 2.8.5. Cell-Associated Exopolysaccharide (EPS) Production

*B. vallismortis* E4 was first qualitatively tested for cell-associated EPS production using aniline blue agar medium. It was then grown for production of cell-associated EPS in MSM as described by Prakash et al. [47]. The inoculum was inoculated into MSM as described in Section 2.8.1 and incubated for 48 h at 150 rpm and 30 °C. Following the incubation period, the medium was centrifuged at 8000 rpm for 20 min, and the pellet was collected for cell-associated EPS extraction.

The pellet was washed with 0.01 M HCl, followed by the addition of 0.5 M NaOH to solubilize the pellet and incubated for 1 h. Subsequently, the suspension was centrifuged at 8000 rpm for 20 min. The supernatant was precipitated with 2.0 M HCl and centrifuged at 8000 rpm. The precipitates were washed with distilled water and dried at 70 °C [47].

##### Measurement of Cell-Associated EPS Viscosity

The viscosity of the samples was measured using a Discovery HR-1 hybrid rheometer (TA Instruments, New Castle, DE, USA). The instrument comprised two stainless steel plates with a 2 mm gap, in which the sample was placed. The parallel-plate geometry, which utilized a 40 mm diameter upper plate with a lower Peltier-controlled plate, was optimized at 25 °C for the viscosity measurements.

##### Characterization of Cell-Associated EPS

To determine the crystalline nature and nanoscale dimensions of cell-associated EPS, atomic force microscopy (AFM) and X-ray diffraction (XRD) analyses were conducted.

To evaluate the cell-associated EPS at the nanoscale, the sample was dissolved in deionized water, spotted on a mica slide, and dried at room temperature. The samples were analyzed in tapping mode using an AFM (Agilent 5500; Agilent Technologies, Santa Clara, CA, USA) at room temperature. Images were captured and assessed using PicoView 1.2 imaging software.

For XRD, the sample was pressed and smoothed to obtain a homogeneous fine powder. The samples were processed using PHYWE XR 4.0 (PHYWE Systeme GmbH & Co. KG, Göttingen, Germany) and analyzed using the Xpert HighScore Plus 2.1 software. This was further assessed using FTIR spectroscopy (Japan Spectroscopic Co., Ltd., Tokyo, Japan).

### 2.9. Statistical Analysis

All production experiments were performed in triplicate. The results are presented as mean ± standard deviation (n = 3) and were calculated in Minitab 18 (Minitab, LLC, State College, PA, USA). As each assay was performed under a single condition, no inferential statistical tests were applied.

## 3. Results and Discussion

### 3.1. Genus and Species Characterization

Alignment of the 16S rRNA sequence with reference sequences from the NCBI database revealed its relationship to the genus *Bacillus*.

Speciation was based on digital DNA-DNA hybridization (dDDH), percentage average nucleotide identity (ANI), difference in %GC content, ribosomal multi-locus sequence typing (rMLST), and multi-locus sequence typing (MLST) of the core genomes.

**Figure 1:**
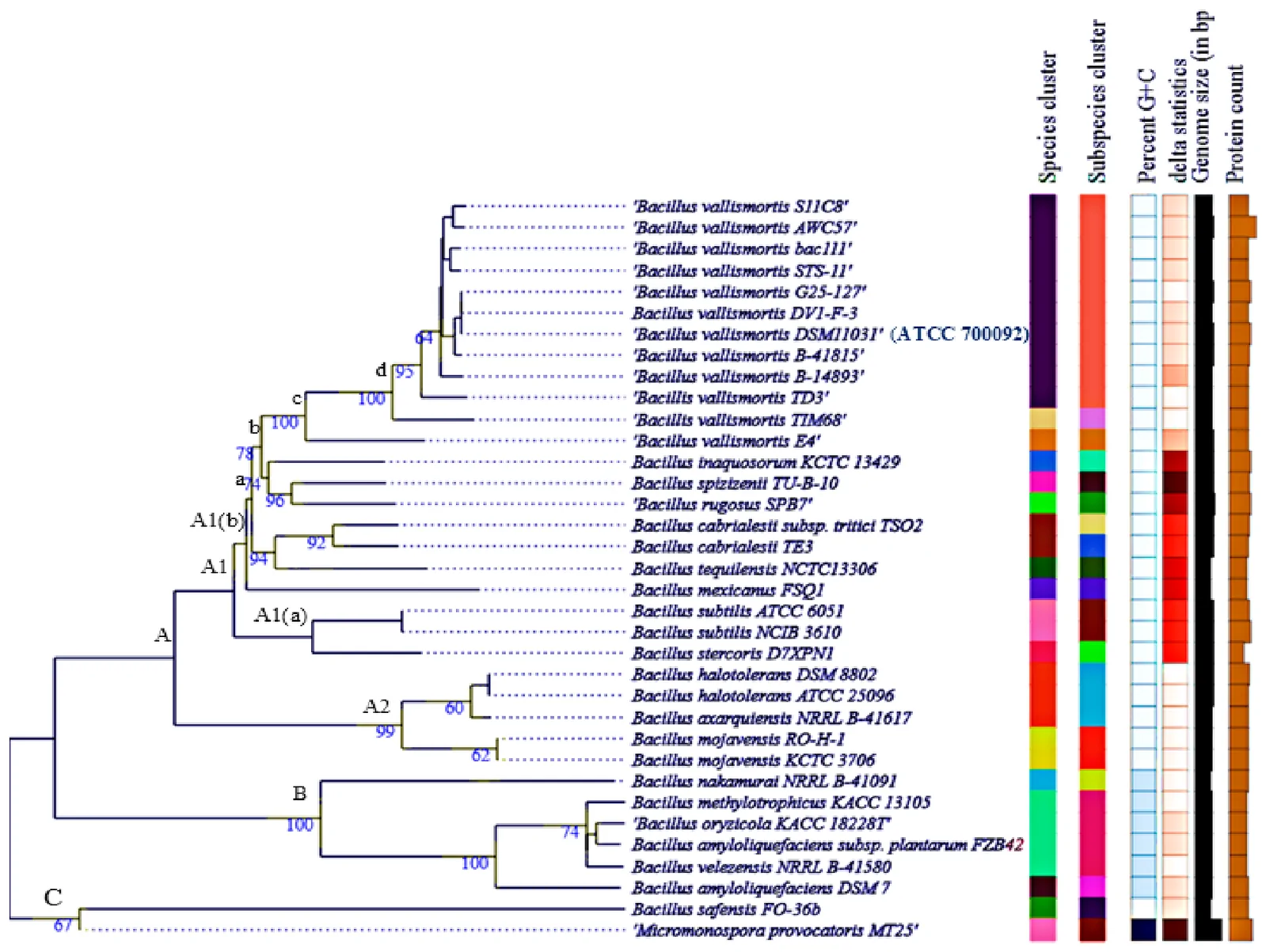
Phylogenomic tree of *Bacillus vallismortis* E4 inferred using FastME 2.1.6.1 from the GBDP distances calculated from genome sequences. Branch lengths were scaled using the GBDP distance formula, *d*_5_. Numbers above the branches are GBDP pseudo-bootstrap support values > 60% from 100 replications, with an average branch support of 57.5%. The tree was rooted at the midpoint.

Interestingly, strain E4 of *B. vallismortis* exhibited intra-strain variations in digital DNA-DNA hybridization (dDDH) values ranging from 77.7% to 84% and in its average nucleotide identity (ANI) percentages when compared to representative genomes. The dDDH value of 77.7% between strain E4 and *B. vallismortis* DSM 11031 (ATCC 700092) (GCF_004116955.1) exceeded the traditional species delineation threshold of≥70%. Although the ANI value of 94.0% was just below the standard of ≥95–96% species boundary, the low G+C content difference (0.09%) fell well within the acceptable intra-species variance limits, supporting its classification within *B. vallismortis*. Despite these variations, G+C content, ribosomal multilocus sequence typing (rMLST), and core genome MLST (cgMLST) analyses confidently classified this isolate as *Bacillus vallismortis*. Phylogenomic reconstruction confirmed that E4 shared the highest genetic similarity with other *B. vallismortis* strains (Figure 1).

Speciation was further confirmed by comparing the dDDH and ANI of our query strain with those of other *Bacillus* species. The dDDH of *B. vallismortis* E4 was 81.8%, 85.7%, 80.4%, 64.8%, 88.6%, 31.3%, and 81.8% similar to *B. inaquosorum*, *B. spizizenii*, *B. subtilis* subsp. *subtilis*, *B. tequilensis*, *B. mojavensis*, *B. amyloliquefaciens,* and *B. velezensis*, respectively; however, the GC content of E4 differed markedly from these species. The ANI of the reported species was 94% with *B. inaquosorum*, 93.7% with *B. spizizenii*, 92.4% with *B. subtilis* subsp. *subtilis*, 92.2% with *B. tequilensis*, 88.6% with *B. mojavensis*, 83.7% with *B. atrophaeus*, 81.9% with *B. amyloliquefaciens*, and 81.8% with *B. velezensis.* Hence, the analyses showed that strain E4 did not closely resemble other *Bacillus* species.

rMLST indexes the genes encoding ribosomal protein subunits. These genes are highly conserved and contain sufficient variation to distinguish between species. rMLST identified 34 ribosomal protein loci (36 alleles, as *rpsN* and *rpmG* were each present in two copies), many of which matched the alleles of *B. vallismortis* (Table S1).

The MLST of the core genome was similar to that of *B. vallismortis*. Eighty housekeeping genes were identified in the MLST of the core genome that encoded proteins, DNA, and protein metabolism, and biosynthetic pathways, constituting the draft genome of *B. vallismortis* E4 (Table S2). The core genome sequence of *B. vallismortis* E4 was used to construct a phylogenetic tree by comparative analysis of its core genome with the up-to-date bacterial core genes (UBCG) set of the reported *Bacillus* spp. The genomes selected for the alignment had fewer differences in G+C content, genome size, and number of proteins, as illustrated in the phylogram (Figure 1). The phylogram illustrated three clades (A, B, and C) arising from the root, and the distance of *B. vallismortis* E4 from the root was 0.1011. Phylogenetic analysis revealed an alignment of *B. vallismortis* E4 with *B. vallismortis* TIM68 (GCF_023953395.1), with evolutionary distance and branch lengths of 0.04 and 0.02, respectively. The branch length shows the divergence of the studied strain from the closely related species during alignment, while the evolutionary distance shows the degree of evolution of the studied strain from the ancestor. *B. vallismortis* E4 belonged to subclade (d) of clade A1(b) (subclade A), while subclade A1(a) harbored *B. subtilis* ATCC 6051 and *B. subtilis* NCIB 3610, indicating their ancestral relationship. Phylogenetic relation of *B. vallismortis* with the *B. subtilis* is coherent with the previous report by Roberts et al. [48], that narrated the close relatedness of *B. vallismortis* and *B. subtilis*. Collectively, the MLST of the core genome, G+C content, and rMLST data indicated that strain E4 was *B. vallismortis*. Its physicochemical characteristics, such as being Gram-positive, rod-shaped, spore-forming, mucoid wrinkled colonies, EPS formation, and surfactin, plipastatin, and iturin production, also support its affiliation with *B. vallismortis* [49]. Thus, it was identified as *B. vallismortis*, as it was found to be closest to *B. vallismortis* compared to other *Bacillus* species.

### 3.2. Specification of the Draft Genome

**Figure 2:**
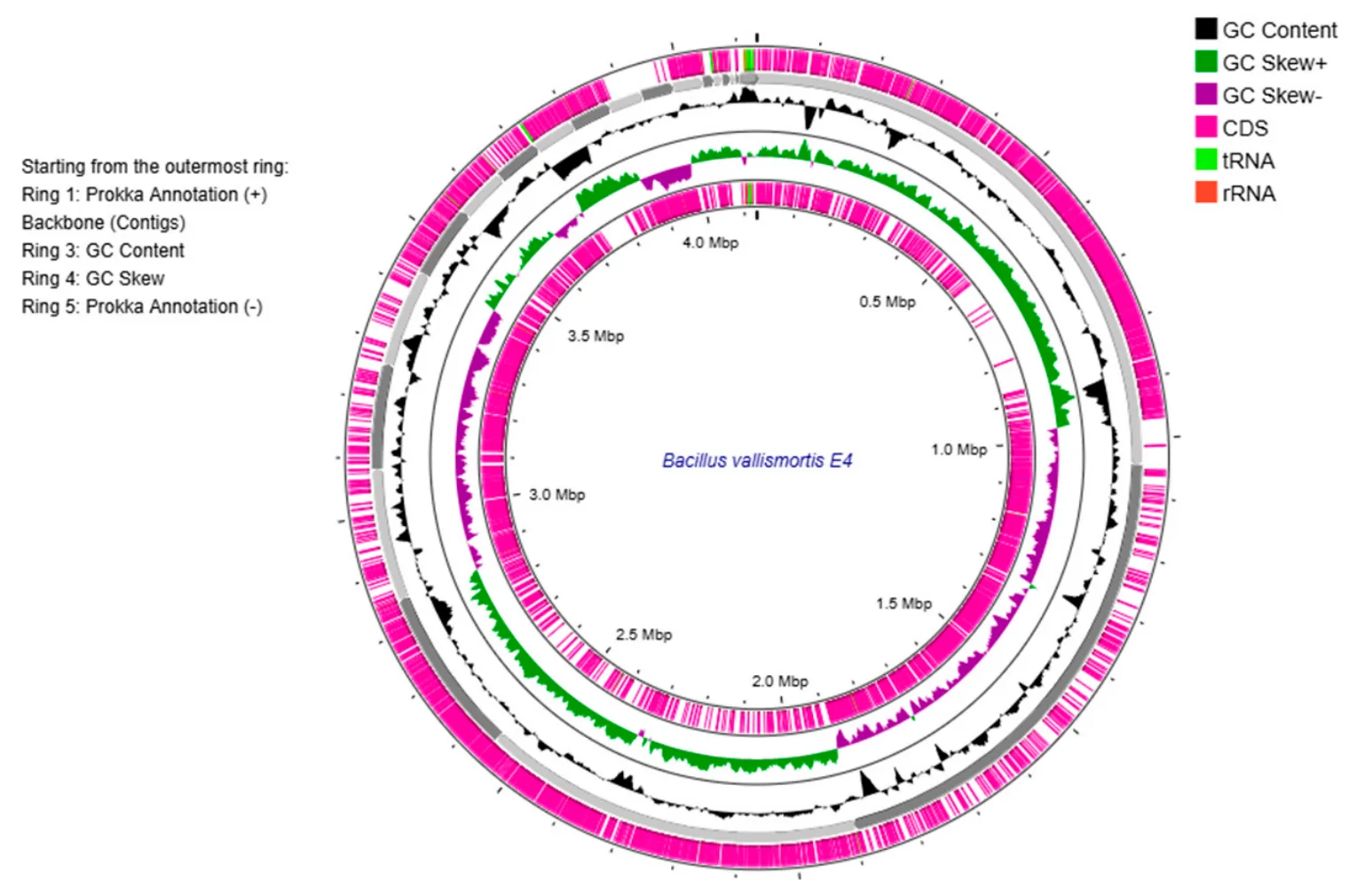
Circular map of the draft genome of *B. vallismortis* E4 from the outer to inner side, representing the forward coding sequence and RNA, contigs, GC content, GC skew, and coding sequence of the reverse strand with RNA genes.

The estimated genome length of *B. vallismortis* E4 was 4,128,106 bp, distributed across 76 contigs, with an average GC content of 43.83 mol%. The sequence length of the shortest contig, at the sum of all contigs that constitute 50% of the total assembly length, that is, N50, was 661,994 bp, while L50 was three contigs that represented half the length of the draft genome (Table 1). A circular graphical display of the distribution of genome annotations from the outer to inner rings is provided in Figure 2, which includes the contigs, CDS on both strands, RNA-encoding genes, GC content, and GC skew.

Genomic annotation of *B. vallismortis* E4 was performed using the NCBI Prokaryotic Genome Annotation Pipeline (PGAP). The PGAP predicted 4167 unambiguous genes, of which 4084 coding sequences were characterized. Of the total number of coding genes, 3943 genes potentially encoded different proteins, whereas 83 genes were RNA-encoding genes (71tRNAs, 5ncRNAs, and 7rRNAs). There were four, two, and one genes encoding 5S, 16S, and 23S rRNA, respectively. The genome of *B. vallismortis* E4 retained 141 pseudogenes without any ambiguous residues and contained 83 frameshift sequences, of which 75 were incomplete, 21 possessed internal stop codons, and 36 exhibited multiple problems. In contrast, Chebotar et al. [22] reported 94 genes of RNA (80 tRNAs, 5 ncRNAs, and 9 rRNAs) and 129 pseudogenes. The differences in strain and source of isolation can be linked to these different gene numbers. The coverage depth, completeness, contamination, and genome quality metrics are listed in Table S3.

Duarte et al. [50] reported a genome size of 3,938,504 bp with 43.89% GC content in the form of 36 contigs with 3912 genes in the draft genome of *B. vallismortis* TIM68. The difference in genome size between *B. vallismortis* TIM68 and the reported strain E4 may be attributed to accessory genome variation, which may include the presence of prophages, mobile genetic elements, or strain-specific genes. It is a common phenomenon among bacterial strains, where the core genome remains conserved and clustered phylogenetically, whereas the accessory genome exhibits variation in size [51,52]. Nonetheless, the GC content of *B. vallismortis* E4 was in alignment with that of the reported *B. vallismortis* strains.

*B. vallismortis* strains have been reported to produce several enzymes. These enzymes are commercially important for animal food processing, stain removal in the detergent industry and bioremediation. Recognizing this species as a biocontrol agent, this study aimed to investigate *B. vallismortis* E4 at the genomic level to understand its role in plant growth promotion by alleviating different stress conditions.

### 3.3. Functional Annotation and Subsystem Analysis

Using PATRIC, the functional annotation of the gene represented 715 hypothetical proteins and 3508 proteins with functional assignments (Table S4). The proteins with functional assignments included 1045 proteins with Enzyme Commission (EC) numbers, 869 with Gene Ontology (GO) assignments, and 766 mapped to KEGG pathways. PATRIC analyzes the proteins in the existing genomic database, and its annotation specifies the genus-specific and cross-genus protein families, PLFams, and PGFams, respectively. The genome contained 3900 PLFams and 3941 PGFams.

PATRIC also indicates the presence of subsystems that are specific to each genome and the set of proteins that constitute a biological process or structural complex. Figure S1 shows a pie chart representing the functional annotation of genes responsible for metabolism, nutrient transport, and the synthesis and degradation of complex molecules. Subsystem analysis revealed the highest number of genes (283) involved in amino acids and their derivatives, followed by 225 genes for carbohydrates and 185 for protein metabolism. Major subsystems included respiration (38 genes), stress response (45 genes), virulence, disease, and defense mechanisms (39 genes), dormancy, and sporulation (97 genes). Moreover, 72 genes from the genome of *B. vallismortis* E4 were involved in DNA replication and cell division, 45 genes were involved in the transport of molecules across the membrane, and 53 genes were associated with RNA processing. In addition to these genes, 78 genes were encoded for the cell wall and cellular envelope (extracellular polysaccharide), 25 genes were responsible for regulation and cell signaling, and 23 genes had unknown functions (Figure S1).

### 3.4. Presence of Genes Indicating Plant Growth Supporting Attributes

The adaptation mechanisms of bacteria that help them resist salinity stress include ion homeostasis, production of universal stress proteins, and accumulation of osmolytes. Transmembrane proteins present in the plasma membrane that maintain homeostasis (cell shape and intracellular pH) are called antiporters. Unlike the soil-resident *B. subtilis* and *B. vallismortis* species, *B. vallismortis* E4 possesses a specific set of halotolerance genes, indicating its adaptation to high-salinity environments. The presence of genes encoding antiporters in the genome of *B. vallismortis* E4, such as *nha*C, *mdr*P-1, (*kht*U, *kht*T-1, khtS), and *kef*B that code for Na^+^/H^+^ antiporter, (Na^+^ Li+ K^+^)/H^+^ antiporter, K^+^/H^+^ antiporter (subunits and modulator), and glutathione-regulated potassium efflux system, respectively, indicated its halotolerant behavior (Table 2). *nha* is a common indicator gene of salt tolerance and homeostasis mechanisms found in PGPB. Chaperones also play an important role in the conservation of stress-affected proteins by maintaining the folding and refolding capability and are upregulated under osmotic stress [53]. Salinity stress is also controlled by the upregulation of abscisic acid, although it has not been studied extensively.

The genome of E4 also harbored the genes *grp*E, *dna*J, and *dna*K (as mentioned in Table 2), which encode heat shock proteins and the regulator gene of these proteins in the form of the heat-inducible transcription repressor *hrc*A. Similar genes have also been reported in different *Bacillus* spp. [54]. In addition to heat shock proteins, the presence of the *yji*B gene in its genome, which encodes a putative cytochrome P450, indicates that it may facilitate the plant under different stress conditions (drought, ultraviolet radiation, salinity, and oxidative stress). The cytochrome P450 also promotes proliferation and controls a variety of secondary metabolites [55]. In addition, the genome of *B. vallismortis* E4 highlighted that this organism can mitigate oxidative and salinity stress in plants by the expression of genes encoding putative manganese catalase (*ydb*D_1), alkyl hydroperoxide reductase (*ahp*C_1), organic hydroperoxide resistance proteins, and its transcriptional regulators (*ohrA, ohrB,* and *ohr*R), flavohemoprotein (*hmp*), heme chaperone, heme-degrading monooxygenase (hmoB), and putative heme-dependent peroxidase (Table 2). Heme-dependent genes reduce oxidative stress directly or indirectly by regulating heme levels. In parallel with these genes, the genome of *B. vallismortis* E4 also demonstrated the presence of glutathione synthetase (*gsh*B) and manganese efflux system protein (*mne*S_1) encoding genes that regulate the levels of glutathione and manganese to prevent plant cell damage. Similarly, other studies have documented the presence of stress-relieving genes such as *hmp, ydbD_1,* and *ahp*C_1 in *Bacillus* sp. BH32 [56]. In this way, PGPB protects the plant through different direct and indirect mechanisms, including nutrient availability, ion homeostasis, modification of root morphology, modulation of stress-responsive genes, synthesis of volatile compounds, exopolysaccharide (EPS), and phytohormones, and aggregation of compatible solutes and antioxidants [57].

Macro- and micronutrients are important for plant growth and survival, in addition to soil requirements. Nitrogen is a macronutrient that constitutes the building blocks of proteins, nucleic acids, and many other molecules. It is also a part of the chlorophyll molecule, which is directly linked to photosynthesis and plant survival [58]. As the nitrogen content is insufficient for plants in saline soil, the role of PGPB is crucial under such conditions. Correspondingly, the genome of *B. vallismortis* E4 also revealed its genomic potential for nitrogen transformation, as it harbors some putative genes of reductases and ureases (*nrg*B, *nmo*A, *yfk*O, *ydj*A, *mhq*N, *yod*C_1, *ure*C, *ure*B, and *ure*A). Urease genes are responsible for the breakdown of urea into ammonia and carbon dioxide. Reductases monitor nitrogen availability in organisms, transform aliphatic nitrogen compounds into nitrites or ammonium, and remove toxic nitrogen compounds, such as the conversion of aromatic nitrogen compounds into simple forms. These genes suggest a potential role in nitrification, ammonia formation, and mineralization, as reviewed by Zamanzadeh-Nasrabadi et al. [59]. Theoretically, the strain has the capacity to fulfill the nitrogen requirement of the plant as an endophyte. However, phenotypic validation is required to confirm the nitrogen fixation potential of this organism.

Following nitrogen, phosphorus is the second macronutrient required by plants, and only 4% of phosphorus is available to plants from the soil. Phosphate-solubilizing bacteria play a vital role in fulfilling the phosphorus requirements of plants by providing them in a simple and accessible form [60]. The genetic makeup of *B. vallismortis* E4, bearing alkaline phosphatase (*pho*A_1 and *pho*A_2) genes, indicates its genetic potential for phosphate solubilization and acquisition by plants. These enzymes convert organic phosphates into inorganic phosphates (Pi), which can be utilized by cells. This needs to be biochemically validated for agricultural applications in the future.

Moreover, the genome of *B. vallismortis* E4 also showed sulfate-metabolizing genes (*cys*P, *sat*_2, *cys*C-2), indicating its genomic capacity to provide the required form of sulfates to the plant, as sulfur is the fourth-most-required macronutrient (Table 2). The *cys*P gene encodes a protein that binds to inorganic sulfate and transports it to the cell. Inorganic sulfate is then converted into organic sulfur compounds through *cys*C-2 gene expression. The *sat*_2 gene regulates sulfur requirements through cysteine formation. Our results are consistent with those of Nascimento et al. [61], who reported the presence of alkaline phosphatase genes (*pho*A and *pho*D) and sulfate metabolism-related genes (*cys*PUWA and *ssu*ABCD) in *B. megaterium* STB1.

Indole acetic acid (IAA) plays an important role in tryptophan metabolism, promoting seedling growth, flower development, and embryogenesis, whereas cytokinins aid in shoot formation, root differentiation, and stress tolerance by metabolizing adenosine-based compounds. The *pat*B, *trp*A, *mia*A, *mia*B, and *yvd*D genes were observed in the WGS of *B. vallismortis* E4. These genes are associated with IAA and cytokinin production pathways, suggesting their genomic capacity for producing phytohormones. These traits may help in future studies to test this strain for sustainable plant growth. These genes have also been identified in the genome of *B. megaterium* STB1 [61]. In addition, it possesses a haloalkane dehalogenase gene that may reduce the adverse effects of pesticides on plants.

### 3.5. Genes Associated with Biopolymer and Lipopeptide Production

Furthermore, the genome of *B. vallismortis* E4 was evaluated for the presence of genes encoding biopolymers and lipopeptides. EPS and biosurfactant (lipopeptide) production genes were identified in the draft genome of *B. vallismortis* E4. Biosurfactant- encoding genes *pps*C, *pps*D, *srf*AD, *srf*AC, and srfAB were found in the genome of *B. vallismortis* E4, which are responsible for Plipastatin synthase subunits (C and D) and Surfactin, respectively (Table 3). Duarte et al. [50] also reported biosurfactant genes, namely vallisin (plipastatin-like lipopeptide), surfactin, and mojavensin, in the genome of *B. vallismortis*.

The capability of EPS synthesis was also observed in the genome of *B. vallismortis* E4 in the form of *sac*B, *sac*, and *sacY-1 genes*, which perform the anabolism and catabolism of levan, levan-sucrase activity, and sucrase synthesis operon, respectively. EPS formation requires depolymerization or synthesis pathways of carbohydrates or polysaccharides, depending on the requirements of the bacterial cell. In addition to these genes, the genomic map of *B. vallismortis* E4 also showed the presence of putative polysaccharide biosynthesis genes (*ywq*C_1, *ywq*C_2, *capA*, *capB*, and *cap*C) (Table 4). In the context of endophytes, EPS formation helps bacteria to attract nutrients and maintain osmotic gradients. EPS also enables bacteria to aggregate and adhere to roots and soil. EPS also improves plant growth, physiology, yield, and soil properties.

In contrast, a gene encoding acetyl-CoA C-acetyltransferase was found in the WGS of *B. vallismortis* E4, which is attributed to the formation of PHB. Moreover, its genome contains an alpha-beta fold hydrolase with an active site structurally similar to that of PhaC. Acetoacetyl-CoA is converted to 3-hydroxybutyryl-coA by a reductase. Although no acetoacetyl-CoA reductase was found, a gene encoding 3-hydroxybutyryl-CoA dehydrogenase may perform an analogous function [62]. In this way, monomers are converted into chains of PHB. Occasionally, PHB formation in bacteria is also associated with the β-oxidation cycle of fatty acids. The strain may follow an alternative path by redirecting acetyl-CoA to PHB production using non-canonical thiolases or reductases, as it has extensive fatty acid metabolism [62]. These may include phospholipases, spore germination lipases, and esterases, which provide a constant supply of fatty acid chains and acetyl-CoA. Nonetheless, transcriptomic investigations are required to formulate hypotheses in this regard.

### 3.6. Detection of Lipase Encoding Genes

Plant cell wall hydrolyzing enzymes, including lipases, aid endophytic bacteria in colonizing the host, nutrient acquisition, and inhibiting plant pathogens [63]. Lipase production was previously recorded in *B. vallismortis* E4 [64]. Genomic mining also validated the existence of genes involved in the degradation of lipids and fatty acids. These genes encode two spore germination lipases (*lip*C1 *and lip*C2), phospholipase, glycerophosphodiester phosphodiesterase, putative phosphoesterase, putative esterase, para-nitrobenzyl esterase, and putative metallophosphoesterase (Table 5). Similarly, phospholipase, spore germination lipase, and carboxyl esterase, phospholipase D, glycerophosphoryl diester phosphodiesterase, and triacylglycerol lipase encoding genes have been reported in the genome of *B. safensis* [65].

### 3.7. Genes Encoding Different Carbohydrases

Different carbohydrate-degrading enzymes, such as cellulases, pectinases, and xylanases, facilitate endophyte colonization and nutrient acquisition. These enzymes are produced by endophytes to establish their presence inside plant tissues. The production of amylase, xylanase, pectinase, and cellulase has been reported in *B. vallismortis.* Genome profiling also supports these findings. Genes encoding endo-1,4-β-xylanase, glucuronoxylanase, arabinoxylan arabinofuranohydrolase, α-amylase, 1,4-α-glucan branching enzyme, glucose-1-phosphate adenyltransferase, glycogen synthase, glycogen phosphorylase, endoglucanase, β-glucanase, aryl-phospho-β-D-glucosidase, oligo-1,6-glucosidase, putative oligo-1,6-glucosidase 2, maltose-6′-phosphate glucosidase, 6-phospho-β-glucosidase, unsaturated rhamnogalacturonyl hydrolase, and pectin lyase were observed in the genome of *B. vallismortis* E4 (Table 6). These genes indicate the potential capacity of *B. vallismortis* E4 to hydrolyze complex carbohydrates. This capability could facilitate the availability of simple compounds to plants that are required for different pathways, such as EPS synthesis (Table 4). In correspondence with these results, different carbohydrases such as endoglucanase, xylanase, α-amylase, pectin lyase, and xylan 1,4-β- xylosidase encoding genes have been mentioned in previous studies by *B. velezensis* CL-4 [66].

### 3.8. Genome-Based Safety Assessment

The presence of antibiotic resistance genes in the genome of *B. vallismortis* E4 poses a concern regarding its further application in the environment or industrial production facilities. However, such intrinsic resistance does not confer the risk of horizontal gene transfer, as it is not plasmid-borne. The presence of these genes indicates that this strain should be used cautiously for environmental applications. Moreover, PathogenFinder2 declared *B. vallismortis* E4 as non-pathogenic to humans (Figure S2). Alignment of the genome with the AMRFinderPlus database, ResFinder, and Comprehensive Antibiotic Resistance Database (CARD) with a >95% similarity index cutoff revealed nine genes that conferred antibiotic resistance (Table 7). The possible role of these genes likely represents the ecological adaptation of organisms to the competitive microenvironment of *A. macrostachyum* and high salinity levels. However, the determination of these antibiotic resistance markers necessitates rigorous safety assessments in humans.

The genome of *B. vallismortis* E4 harbored genes associated with intrinsic resistance via antibiotic-inactivating enzymes, putative antibiotic target protection proteins, target replacement proteins, efflux pumps, and alteration of cell wall charge through protein formation that prevents antibiotic binding, and through regulators that modulate the expression of antibiotic resistance genes. Its genome possessed *bsu* and *mph*K genes that inactivate antibiotics through β-lactamase enzymes, penicillin-binding proteins and macrolide 2 phosphotransferases that confer resistance to carbapenem, ampicillin and macrolides, respectively [67,68]. Another *ykkCD* gene, which encodes a multidrug efflux pump, was also detected in its genome that expels toxicants by binding to tetracycline [69]. The presence of *mpr*F genes into the genome of *B. vallismortis* E4 may confer resistance to daptomycin by altering cell wall charge [70]. The genome of *B. vallismortis* E4 also exhibited *lmr*B responsible for the resistance to different drugs including lincomycin and bacitracin. The genome also possessed *tmr*B and *bmr* genes that alleviate the effect of tunicamycin and fluoroquinolones, acridine dyes (cationic antimicrobials) by modulating the cell wall permeability and its efflux mechanism, respectively [71,72]. The E4 genome also exhibited *vml*R gene that prevents the binding of lincosamide, streptogramin type A and pleuromutilin to ribosome and protects the translation process [73]. According to European food safety authority (EFSA) [74], evaluation of antibiotic resistance against eight antibiotics have been recommended to declare it as safe for human consumption. Of these antibiotics, we identified three antibiotic resistance genes, although their phenotypic resistance needs confirmation through minimum inhibitory concentration (MIC) determination, as it is possible to have MIC values less than the mentioned concentration of these antibiotics by EFSA.

### 3.9. Presence of Genes for Secondary Metabolites

AntiSMASH predicted different secondary metabolite genes in the genome of *B. vallismortis* E4, such as genes for antibiotic production (subtilosin A, bacilysin, and bacillaene), an antifungal lipopeptide (fengycin), iron-chelating compounds (bacillibactin and pulcherriminic acid), and antioxidant compounds (sactipeptides). Our results are in accordance with those of Chebotar et al. [22], who also reported different antimicrobial metabolite genes in *B. vallismortis BL-01*, including bacillaene, fengycin, bacillibactin, bacilysin, and subtilosin A.

### 3.10. Experimental Corroboration for the Biopolymers and Lipopeptides Production

Because biopolymers and lipopeptides are biodegradable, their microbial production is environmentally friendly and sustainable. Therefore, we experimentally tested whether the genes predicted in strain E4 were reflected in actual production.

#### 3.10.1. Lipopeptide (Biosurfactant) Production

In addition to cell-associated EPS potential, the endophytic strain was further screened for biosurfactant production using MSM with ground CF seeds as a complex carbon source. The biosurfactant produced was collected through acidification of the post-fermentation hydrolysate and quantified as 1 mg mL^−1^. The cell-free culture supernatant was tested as a crude biosurfactant for oil spreading ability in Petri dishes, and biosurfactant activity was observed (Figure S3A). The oil spreading test is a rapid, reliable, and convenient method for analyzing biosurfactant production.

The oil displacement area of 36.65 ± 0.38 cm^2^ was determined for biosurfactant using burned diesel oil, which was considerably larger than those reported for *B. rugosus* DTS1 and *B. subtilis* DTS2 (2.0 and 2.1 ± 0.1 cm^2^) [75]. However, differences in oil type, sample volume, and dish size limit direct comparison.

The biosurfactant activity was further confirmed using an emulsification assay. The ability to form emulsions with hydrocarbons is important for bioremediation, oil tank cleaning, and the treatment of industrial effluents. Emulsion formation increases the miscibility of two immiscible liquids by reducing the surface and interfacial tensions of the liquids [76]. The emulsifying characteristics of the samples were analyzed by determining the ratio of the height of the emulsion to that of the suspension. The suspension comprised oil and a sample with emulsification properties. Olive oil, a hydrophobic substrate, was used in the assay. The emulsification index of the biosurfactant produced by *B. vallismortis* E4 was 49.66 ± 3.51% (Figure S3B). Previously, a maximum emulsification index of 75.1% was recorded after 24 h by *Paenibacillus* sp. using crude oil [77].

#### 3.10.2. PHB Formation

The PHB production by E4 strain was investigated in MSM. The strain yielded 5.47 ± 0.12 wt% PHB from 3.35 g/L dry cell weight. In contrast, the PHB production by *B. paramycoides* and *B. subtilis* was reported to be 32.86 wt% and 68.71 wt % of the dry cell weight of 2.67 g/L and 1.79 g/L, respectively [78,79]. The low yield requires optimization to achieve maximum production.

**Figure 3:**
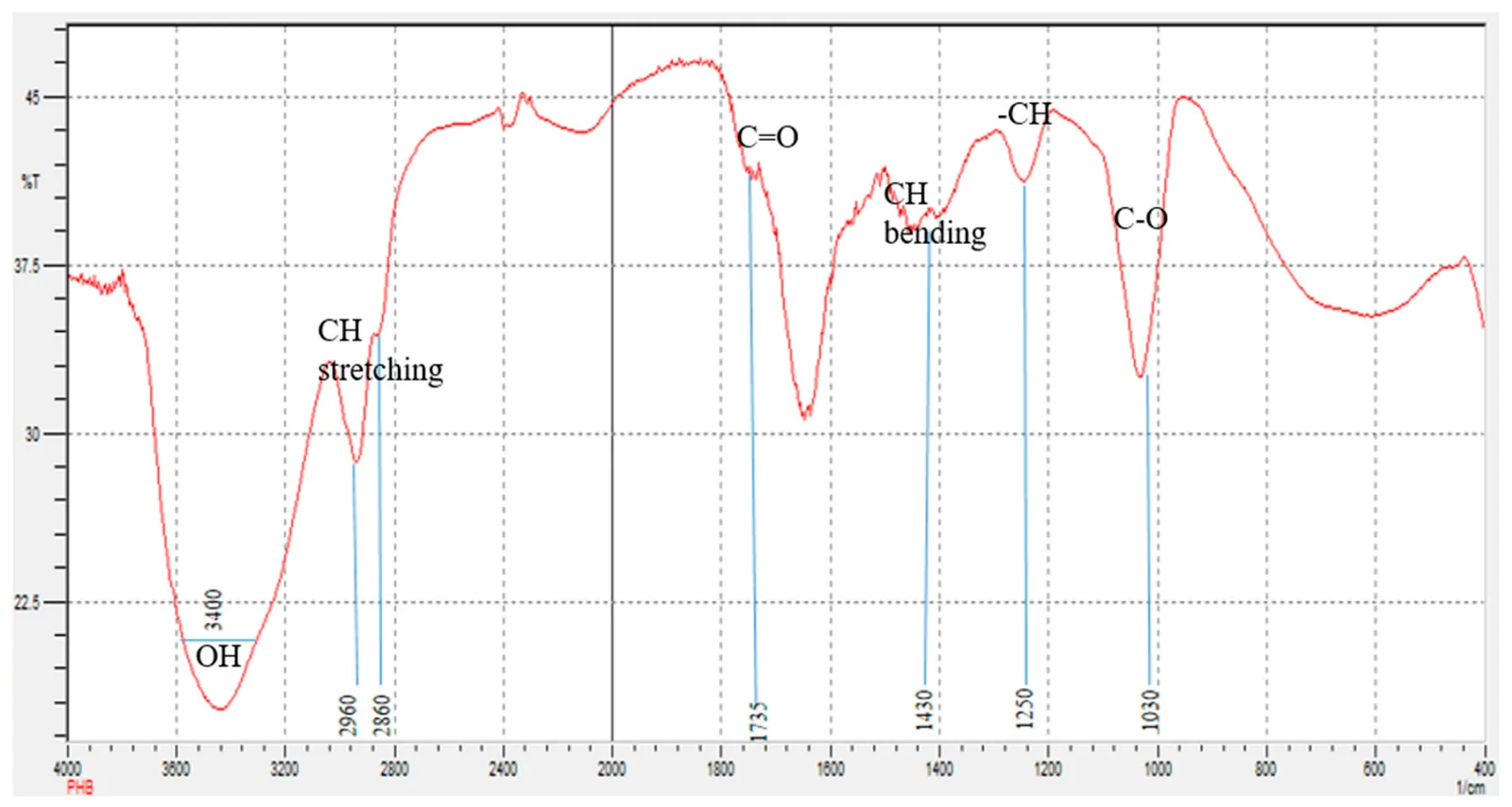
Illustration of the IR pattern of polyhydroxybutyrate from *B. vallismortis* E4.

The FTIR spectrum of PHB showed an absorption band at 1735 cm^−1^ corresponding to the carbonyl group of the ester and another band at 1250 cm^−1^ corresponding to the –CH group; according to Sindhu et al. [80], these bands are considered PHB markers. The peak at 1030 cm^−1^ represents the C-O stretching of the ester group. The vibrations at 2860 and 2960 indicate the presence of CH stretching of the methylene and methyl groups of PHB, respectively, and the CH bending was found between 1370 and 1460. The broad peak at 3400 indicates the terminal OH groups. The vibrations at 1639, 1508–1593, and 1400–1450 were due to C=C stretching, aromatic structures, and aromatic structures along with CH deformation (Figure 3). Based on the FTIR spectra, the sample was preliminarily identified as PHB.

**Figure 4:**
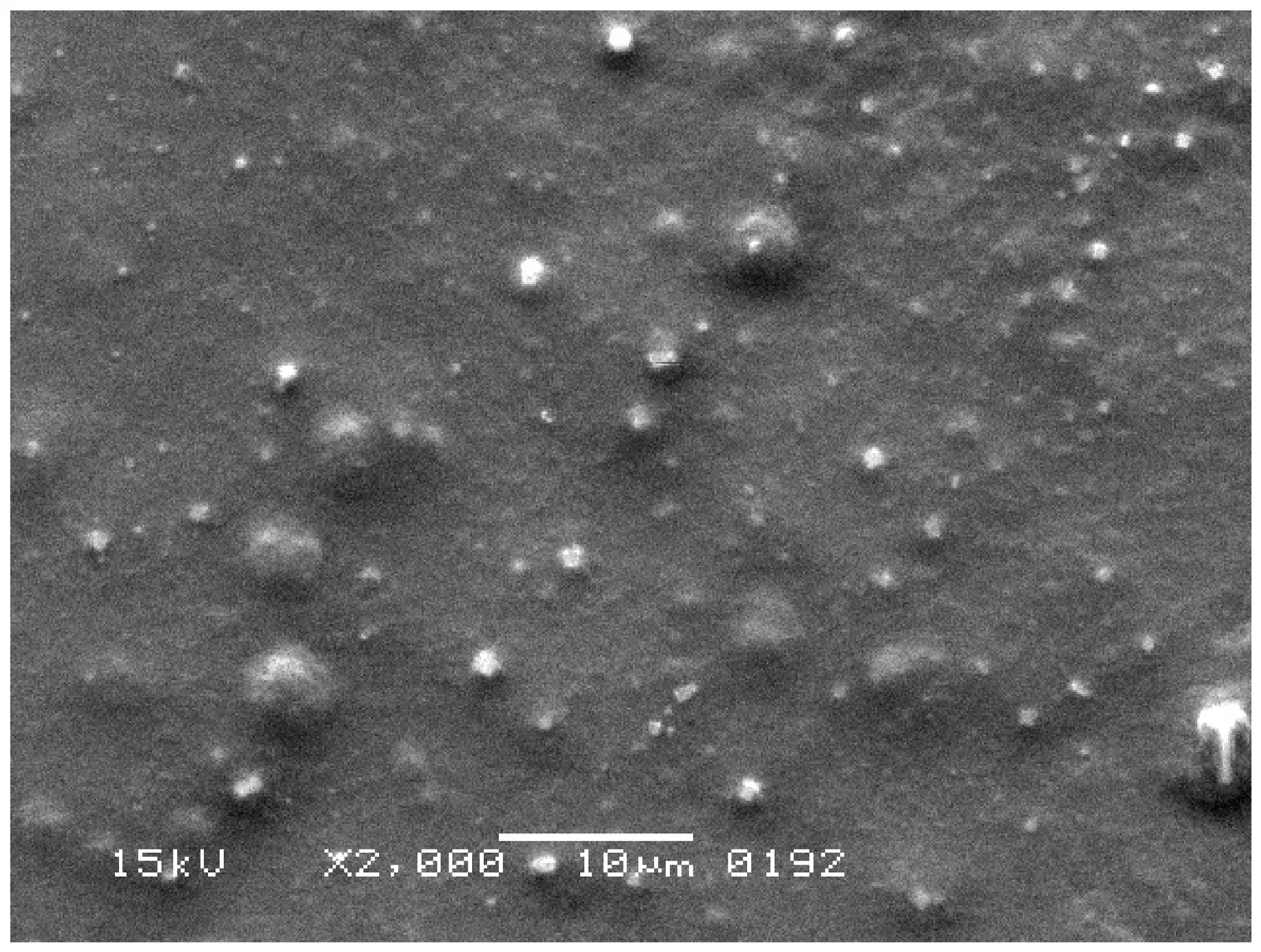
SEM image of polyhydroxybutyrate produced by *B. vallismortis* E4.

The surface morphology of PHB was examined using SEM. The surface was slightly rough, indicating that the microstructure appeared spherical (Figure 4) [81].

#### 3.10.3. Production of Cell-Associated EPS

**Figure 5:**
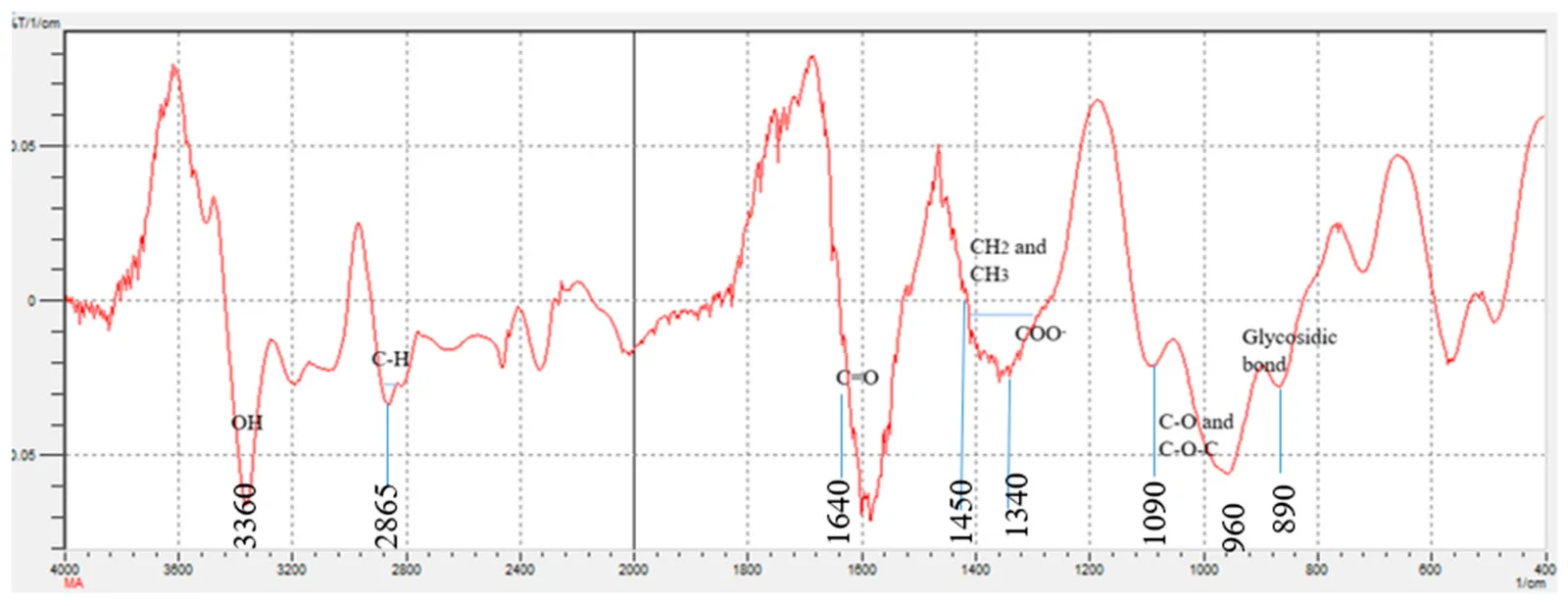
IR spectra of cell-associated exopolysaccharides produced by *B. vallismortis* E4, elucidating notable peaks.

EPS-producing plant growth-promoting bacteria stimulate plant growth under drought conditions by adhering to clay particles, leading to soil aggregation. This improves root and shoot growth by modifying the soil structure, resulting in an expanding root system in the plant and improved water and nutrient uptake for prolonged hydration. In addition to these properties, it also nullifies the adverse effects of reactive oxygen species [82]. Considering its importance and vital role in plant growth, the E4 strain was assessed for cell-associated EPS production in the presence of a carbon-supplemented medium and extracted through precipitation. The yield was 35.3 ± 0.1 mg/100 mL cell-associated EPS, which was further analyzed using FTIR, AFM, and XRD. The spectroscopic data, as shown in Figure 5, showed notable peaks at 890 cm^−1^ of the β-glycosidic bond or β-D-glucan and the absorption peak at 3360 cm^−1^, representing the bonded OH stretch and indicating its polysaccharide nature [83]. The absorption band at 570 cm^−1^ showed the existence of a glycosidic linkage between the glycosyl groups of the compound [84]. The stretching at 1360 cm^−1^ indicated the presence of a carboxyl group in the polymer that can serve as a binding site for divalent ions. The presence of carboxyl and hydroxyl groups could be responsible for the mucous and viscous nature of the polymer [84]. Next, the bending vibrations of CH_2_ and CH_3_ at 1340 cm^−1^ and 1450 cm^−1^ and the stretching bands of C-H of aliphatic hydrocarbon at 2865 cm^−1^ have been observed. The stretching peaks of C-O and C-O-C at 960 and 1090 cm^−1^, indicating the presence of carbohydrate, and C=O at 1640 cm^−1^ were noted [85]. The C-O band confirms the presence of the polymer.

**Figure 6:**
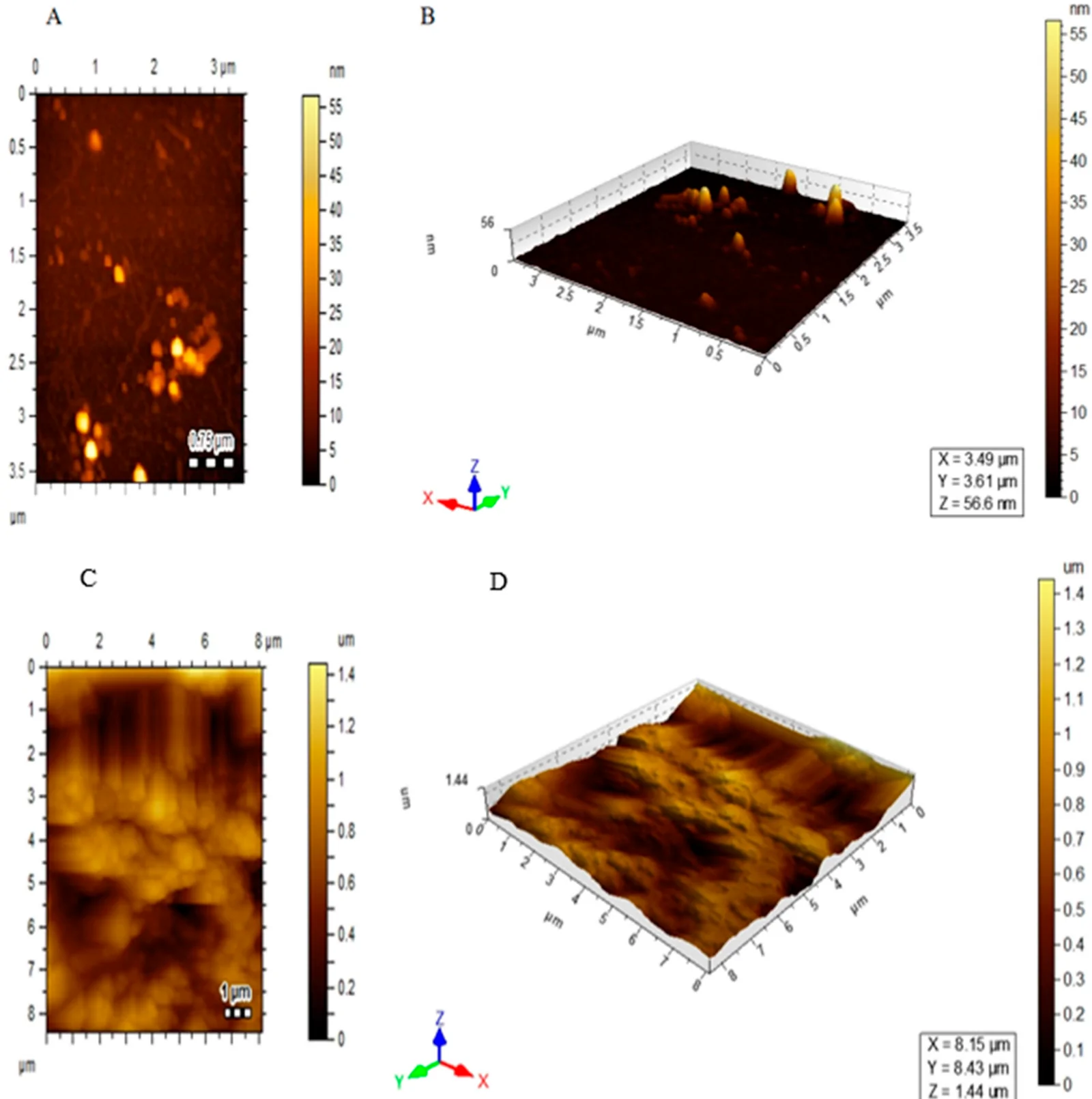
AFM images of cell-associated EPS from *B. vallismortis* E4. (**A**) Two-dimensional height image and (**B**) three-dimensional view of a 3.5 × 3.6 µm scan (Z range: 0–56 nm). (**C**) Two-dimensional height image and (**D**) three-dimensional view of an 8.2 × 8.4 µm scan (Z range: 0–1.44 µm).

AFM was used to visualize the morphological parameters at the nanoscale, including the surface roughness, particle size, height, and width of the sample. The topological properties of polysaccharides have been widely characterized using AFM [86]. In recent studies, AFM has been used to characterize and understand the dynamics of polymers, macromolecular conformations, and their topographical features [87]. Round lumps were observed, indicating the irregular and rough surface of the cell-associated EPS, which had a height ranging from 30 to 51 nm and an average particle size distribution of 31.14 nm (Figure 6A,B).

The hydrophilic nature of the polysaccharide may contribute to its viscosity. Their affinity to water molecules may contribute to a substantial viscous nature and superior gelling property [87]. However, conclusions should be drawn cautiously, as the drying process during preparation may affect the pattern observation. The presence of round lumps at the nanoscale and the high affinity of water molecules suggest a potential for shear-dependent flow characteristics. The molecular conformation of the polymer resembles the tertiary structure of curdlan as reported by Nishinari et al. [88] (Figure 6C,D). These conformations suggest that the extracted cell-associated EPS molecule could be curdlan; however, it requires monosaccharide composition analysis, NMR, and comparison with a curdlan standard. The characteristic features observed in the AFM images were also supported by the rheological properties.

The cell-associated EPS exhibited an apparent viscosity of 0.099 ± 0.006 Pa.s in a 1% solution, highlighting its good gelling property and viscous nature, which is consistent with its morphology. In contrast, Jakhu et al. [89] noted a lower viscosity of 0.04 Pa.s for 4% EPS. These results suggest that the viscosity may vary with respect to the composition of the polymer, which contributes to its viscosity.

**Figure 7:**
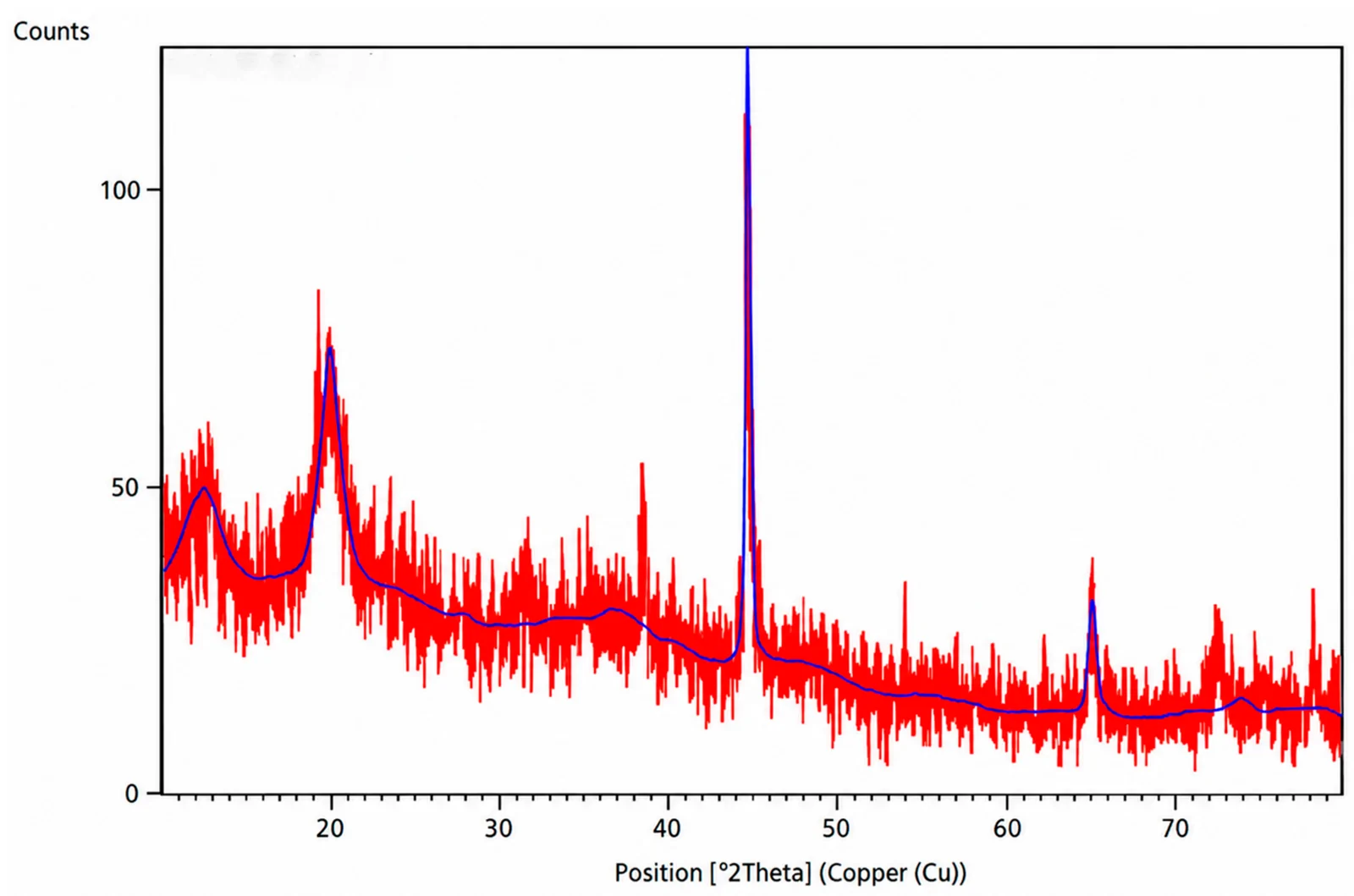
XRD patterns of cell-associated EPS collected from *B. vallismortis* E4.

XRD was employed to determine the nature of the compound and its crystallinity. The XRD pattern of cell-associated EPS (Figure 7) exhibited different sharp peaks from 0 to 70 spectra in the 2θ position. The intense narrow peaks at 12.93, 21.00, 26.80, 31.50, and 44.70 indicate the crystalline nature of the compound, while the peaks at 10.79 and 19.36 represent the polysaccharide polymer (Figure 7). Our results are consistent with those of Prakash et al. [47].

## 4. Conclusions

In this study, we present the whole-genome sequence of *B. vallismortis* E4, which comprises core genome analysis, genome, and functional annotation. Its genetic makeup exhibits genes related to salt tolerance, antioxidant enzymes, heat shock proteins, lignocellulose-degrading enzymes, lipases, sulfur, nitrogen, and phosphate metabolism-regulating genes, biopolymers (PHB and EPS), and lipopeptide-encoding genes. *B. vallismortis* E4 has preliminary PHB-producing potential, which requires optimization and validation. The genomic architecture of *B. vallismortis* E4 shows its significant potential for biopolymer and lipopeptide production and for plant growth promotion. WGS provides a framework for the different traits encoded in DNA. Future physiological and transcriptomic studies are required to unveil their metabolic contributions to the host environment. Subject to further biosafety assessment, strain E4 may be suitable for environmental applications, such as bioremediation, plant growth promotion, and biocontrol.

## Figures and Tables

**Table 1:** Statistics of the genome of *Bacillus vallismortis* E4.

Contigs	76
GC Content	43.83%
Plasmids	0
Contig L50	3
Genome Length	4,128,106 bp
Contig N50	661,944
Protein Coding Sequence	3943
tRNA	71
rRNA	7

**Table 2:** Presence of genes related to different attributes in *B. vallismortis* E4 that assist in plant growth.

Genes	Function	Associated Mechanism
*nha*C	Na^+^/H^+^ antiporter	Involved in pH homeostasis and halo-tolerance
*mdr*P-1	Na^+^, Li+, K^+^/H^+^ antiporter	
*kef*B	Glutathione-regulated potassium-efflux system	
*kht*U, *kht*T and *Kht*S	Subunits for K^+^/H^+^ antiporter	
*grp*E	Co-chaperone Protein	Stress tolerance (heat shock proteins)
*dna*J	Chaperone protein	
*dna*K	Chaperone protein	
*hrc*A	Heat-inducible transcription repressor	
*yji*B	Putative cytochrome P450	
*hmp*	Flavohemoprotein	Antioxidant
*ydb*D_1	Putative manganese catalase	
*gsh*B	Glutathione synthetase	
*mne*S_1	Manganese efflux system protein	
*ohr*A	Organic hydroperoxide resistance protein	
*ohr*R	Organic hydroperoxide resistance transcriptional regulator	Putative Nitrogen cycling
*ohr*B	Organic hydroperoxide resistance protein	
*ahp*C_1	Alkyl hydroperoxide reductase C	
*yfk*O	Putative NAD(P)H nitroreductase	
*ydj*A	Putative NAD(P)H nitroreductase	
*mhq*N	Putative NAD(P)H nitroreductase	
*ure*C	Urease subunit alpha	Ammonia metabolism
*ure*B	Urease subunit beta	
*ure*A	Urease subunit gamma	
*cys*P	Sulfate permease	Sulphur metabolism
*sat*_2	Sulfate adenyltransferase	
*cys*C_2	Putative adenyl-sulfate kinase	
*pho*A_1	Alkaline phosphatase 4	Phosphate solubilization
*pho*A_2	Alkaline phosphatase 4	
*pat*B	Cystathionine beta-lyase	Indole Acetic Acid
*trp*A	Tryptophan synthase alpha chain	
*mia*A	tRNA dimethylallyltransferase	Cytokinin production
*mia*B	tRNA-2-methylthio-N (6)-dimethyl allyladenosine synthase	
*yvd*D	LOG family protein	

**Table 3:** Presence of lipopeptide (biosurfactant) genes in the genomic makeup of *B. vallismortis* E4.

Gene	Function
*pps*C	Plipastatin synthase subunit C
*pps*D	Plipastatin synthase subunit D
*srf*AD	Surfactin synthase thioesterase subunit
*srf*AB	Surfactin synthase thioesterase subunit 2
*srf*AC	Surfactin synthase thioesterase subunit 3

**Table 4:** Genes potentially involved in polysaccharide and capsule synthesis in *B. vallismortis* E4.

Gene	Function
*sac*B	Levansucrase
*sac*	Levanase
*sac*Y_1	Levansucrase and sucrase synthesis operon
*ywq*C_1	putative capsular polysaccharide biosynthesis protein
*ywq*C_2	putative capsular polysaccharide biosynthesis protein
*cap*A	Capsule biosynthesis protein
*cap*B	Capsule biosynthesis protein
*cap*C	Capsule biosynthesis protein

*sacB* and *sac* are associated with secreted levan, and *capA/B/C* are homologues of *pgsBCA*, which encodes poly γ-glutamate capsule synthesis in Bacillus.

**Table 5:** Genes involved in lipase production by *B. vallismortis* E4.

Genes	Function
*lip*C_1	Spore germination lipase
*est*A	Lipase
*lip*C_2	Spore germination lipase
*ytp*A	Phospholipase
*glp*Q-1	Glycerophosphodiester phosphodiesterase
*yjc*G	Putative phosphoesterase
*yxi*M	putative esterase
*pnb*A	Para-nitrobenzyl esterase
*yha*O	putative metallophosphoesterase

**Table 6:** Presence of carbohydrate-degrading genes in the genome of *B. vallismortis* E4.

Gene	Function
*xyn*A_1, *xyn*A_2	Endo-1,4-β- xylanase A
*xyn*C	Glucuronoxylanase
*xyn*D	Arabinoxylan arabinofuranohydrolase
*amy*E	α-amylase
*glg*B	1,4-α-glucan branching enzyme
*glg*C	glucose-1-phosphate adenyltransferase
*glg*D	Glycogen biosynthesis protein
*glg*A	Glycogen synthase
*glg*P	Glycogen phosphorylase,
*bgl*A	Aryl-phospho-β-D-glucosidase,
ycdG	Putative oligo-1,6-glucosidase 2
*glv*A	Maltose-6′-phosphate glucosidase
*gmu*D	6-phospho-β-glucosidase
*yes*R	Unsaturated rhamnogalacturonyl hydrolase
*pel*B	Pectin lyase
*egl*S	Endoglucanase
*bgl*S	β-glucanase
*bgl*H	Aryl-phospho-beta-D-glucosidase
*bgl*F_1	PTS system beta-glucoside-specific EIIBCA component
*mal*L_2	Oligo-1,6-glucosidase
*bgl*C	Aryl-phospho-beta-D-glucosidase

**Table 7:** The antibiotic resistance genes were similar to those in the AMRFinderPlus database, ResFinder, and the Comprehensive Antibiotic Resistance Database (CARD).

Sequence	Start	End	Strand	Gene	%Coverage	%Identity	Database
NODE_2_length_851500_cov_146.003235	334,560	335,362	+	*bla*_BSU_-1	99.88	95.39	NCBI
NODE_6_length_182779_cov_144.652221	112,115	113,035	-	*mph*K	100	90.88	NCBI
NODE_9_length_86908_cov_152.230321	56,061	57,691	-	*vml*R	98.78	90.32	NCBI
NODE_6_length_182779_cov_144.652221	112,115	113,035	-	*mph*_K-1_	100	90.88	resfinder
NODE_1_length_1045533_cov_144.161280	384,396	384,734	+	*ykk*C	100	90.86	CARD
NODE_1_length_1045533_cov_144.161280	384,734	385,051	+	*ykk*D	100	94.34	CARD
NODE_3_length_661944_cov_143.350956	601,678	602,844	-	*bmr*	99.74	90.77	CARD
NODE_6_length_182779_cov_144.652221	48,042	48,635	+	*tmr*B	100	89.73	CARD
NODE_6_length_182779_cov_144.652221	97,665	99,098	+	*lmr*B	100	90.8	CARD
NODE_6_length_182779_cov_144.652221	112,115	113,035	-	*mph*K	100	90.88	CARD
NODE_8_length_127532_cov_141.673045	96,924	99,494	+	*mpr*F	100	90.31	CARD
NODE_9_length_86908_cov_152.230321	56,061	57,691	-	*vml*R	98.78	90.32	CARD

## Data Availability

The data of the whole genome of *B. vallismortis* E4 is available in the NCBI database under the accession number of JBIMFA000000000.

## Supplementary Materials

The following supporting information can be downloaded at: https://www.mdpi.com/article/10.3390/life16101707/s1, Table S1: Ribosomal protein gene loci identification and their allelic genes for *B. vallismortis* E4 through multi-locus sequence typing of the 16s rDNA genome (rMLST). Table S2: Gene sequence assignments for *B. vallismortis* E4 based on multi-locus sequence typing (MLST) of the genome. Table S3: Genome sequencing quality profile of *B. vallismortis* E4. Table S4: Summary of functional annotation of *Bacillus vallismortis* E4 proteome, showing enzymes (EC), gene ontology (GO), metabolic pathway, and PATRIC family assignments. Figure S1: Pie chart illustrating the metabolic classes of annotated genes present in the draft genome of *B. vallismortis* E4. Figure S2: Figure representing the analysis of pathogenicity of *B. vallismortis* E4 on the basis of neural network principle using PathogenFinder2. Figure S3: Biosurfactant analysis: (A) is exhibiting oil displacement area in the presence of biosurfactant produced by *B. vallismortis* E4, and (B) is showing emulsification of olive oil in the presence of biosurfactant produced by *B. vallismortis* E4.
