# Supplementary material for: Integrated Phenotypic and Genomic Characterization of Endophytic Bacillus vallismortis E4 for Biopolymer and Lipopeptide Production
Source: Life (Basel). 2026 Oct 12;16(10):1707. doi: 10.3390/life16101707 (PMC13706435; doi:10.3390/life16101707)
Supplement: Supplementary file 1 [file life-16-01707-s001.zip › life-4501625-supplementary.pdf]

Table S1. Ribosomal protein gene loci identified in *B. vallismortis* E4 by ribosomal multilocus sequence typing (rMLST).

Locus	Allele	Length	Contig	Start position	End position
BACT000003 (rpsC)	18793	657	NODE_13_length_59949_cov_144.988910	21575	22231
BACT000004 (rpsD)	20494	603	NODE_3_length_661944_cov_143.350956	135948	136550
BACT000005 (rpsE)	17563	501	NODE_13_length_59949_cov_144.988910	17213	17713
BACT000006 (rpsF)	8947	288	NODE_2_length_851500_cov_146.003235	825487	825774
BACT000008 (rpsH)	16048	399	NODE_13_length_59949_cov_144.988910	18702	19100
BACT000011 (rpsK)	16024	396	NODE_13_length_59949_cov_144.988910	12304	12699
BACT000012 (rpsL)	39127	417	NODE_13_length_59949_cov_144.988910	30957	31373
BACT000013 (rpsM)	15745	366	NODE_13_length_59949_cov_144.988910	12720	13085
BACT000014 (rpsN)	24795	270	NODE_12_length_71079_cov_140.904608	6318	6587
BACT000014 (rpsN)	393	186	NODE_13_length_59949_cov_144.988910	19132	19317
BACT000015 (rpsO)	3677	270	NODE_1_length_1045533_cov_144.161280	745839	746108
BACT000018 (rpsR)	5759	240	NODE_2_length_851500_cov_146.003235	824642	824881
BACT000019 (rpsS)	13730	279	NODE_13_length_59949_cov_144.988910	22593	22871
BACT000020 (rpsT)	6006	267	NODE_3_length_661944_cov_143.350956	463269	463535
BACT000021 (rpsU)	1534	174	NODE_3_length_661944_cov_143.350956	478807	478980
BACT000032 (rplC)	40393	630	NODE_13_length_59949_cov_144.988910	24732	25361
BACT000035 (rplF)	18774	540	NODE_13_length_59949_cov_144.988910	18133	18672
BACT000036 (rplL)	17880	372	NODE_13_length_59949_cov_144.988910	40098	40469
BACT000040 (rplK)	18155	426	NODE_13_length_59949_cov_144.988910	42060	42485
BACT000043 (rplN)	16097	369	NODE_13_length_59949_cov_144.988910	20254	20622

BACT000044 (rplO)	17621	441	NODE_13_length_59949_cov_144.988910	16549	16989
BACT000046 (rplQ)	16582	363	NODE_13_length_59949_cov_144.988910	10744	11106
BACT000047 (rplR)	15313	363	NODE_13_length_59949_cov_144.988910	17738	18100
BACT000050 (rplU)	14939	309	NODE_3_length_661944_cov_143.350956	309399	309707
BACT000051 (rplV)	3719	342	NODE_13_length_59949_cov_144.988910	22235	22576
BACT000052 (rplW)	14269	288	NODE_13_length_59949_cov_144.988910	23794	24081
BACT000056 (rpmA)	15466	285	NODE_3_length_661944_cov_143.350956	310061	310345
BACT000058 (rpmC)	11947	201	NODE_13_length_59949_cov_144.988910	20949	21149
BACT000059 (rpmD)	11324	180	NODE_13_length_59949_cov_144.988910	17020	17199
BACT000060 (rpmE)	25666	201	NODE_2_length_851500_cov_146.003235	409117	409317
BACT000061 (rpmF)	737	180	NODE_1_length_1045533_cov_144.161280	582521	582700
BACT000062 (rpmG)	383	150	NODE_3_length_661944_cov_143.350956	523728	523877
BACT000062 (rpmG)	21866	150	NODE_13_length_59949_cov_144.988910	43577	43726
BACT000063 (rpmH)	2944	135	NODE_2_length_851500_cov_146.003235	841341	841475
BACT000064 (rpmI)	95343	201	NODE_3_length_661944_cov_143.350956	208518	208718
BACT000065 (rpmJ)	332	114	NODE_13_length_59949_cov_144.988910	13108	13221

Table S2: Figure S2. PathogenFinder2 prediction output for *B. vallismortis* E4 (screenshot of the results table)

TIGR00133	gatB	Protein synthesis	aspartyl/glutamyl-tRNA(Asn/Gln) amidotransferase, B subunit
TIGR00132	gatA	Protein synthesis	aspartyl/glutamyl-tRNA(Asn/Gln) amidotransferase, A subunit
TIGR03953	rplD_bact	Protein synthesis	50S ribosomal protein uL4
TIGR03534	RF_mod_PrmC	Protein fate	protein-(glutamine-N5) methyltransferase, release factor-specific

TIGR00135	gatC	Protein synthesis	aspartyl/glutamyl-tRNA(Asn/Gln) amidotransferase, C subunit
TIGR00019	prfA	Protein synthesis	peptide chain release factor 1
TIGR00060	L18_bact	Protein synthesis	ribosomal protein uL18
TIGR00647	DNA_bind_WhiA	Cellular processes	DNA-binding protein WhiA
TIGR00138	rsmG_gidB	Protein synthesis	16S rRNA (guanine(527)-N(7))-methyltransferase RsmG
TIGR01978	sufC	Biosynthesis of cofactors, prosthetic groups, and carriers	FeS assembly ATPase SufC
TIGR00059	L17	Protein synthesis	ribosomal protein bL17
TIGR00244		Regulatory functions	transcriptional regulator NrdR
TIGR01146	ATPsyn_F1gamma	Energy metabolism	ATP synthase F1, gamma subunit
TIGR00593	pola	DNA metabolism	DNA polymerase I
TIGR00152		Biosynthesis of cofactors, prosthetic groups, and carriers	dephospho-CoA kinase
TIGR00173	menD	Biosynthesis of cofactors, prosthetic groups, and carriers	2-succinyl-5-enolpyruvyl-6-hydroxy-3-cyclohexene-1-carboxylic-acid synthase
TIGR00767	rho	Transcription	transcription termination factor Rho
TIGR00038	efp	Protein synthesis	translation elongation factor P
TIGR00484	EF-G	Protein synthesis	translation elongation factor G
TIGR00763	lon	Protein fate	endopeptidase La
TIGR00575	dnlj	DNA metabolism	DNA ligase, NAD-dependent
TIGR00981	rpsL_bact	Protein synthesis	ribosomal protein uS12
TIGR01039	atpD	Energy metabolism	ATP synthase F1, beta subunit
TIGR01169	rplA_bact	Protein synthesis	ribosomal protein uL1
TIGR00331	hrcA	Regulatory functions	heat-inducible transcription repressor HrcA
TIGR00096	TIGR00096	Protein synthesis	16S rRNA (cytidine(1402)-2'-O)-methyltransferase
TIGR01044	rplV_bact	Protein synthesis	ribosomal protein uL22
TIGR01302	IMP_dehydrog	Purines, pyrimidines, nucleosides, and nucleotides	inosine-5'-monophosphate dehydrogenase
TIGR00355	purH	Purines, pyrimidines, nucleosides, and nucleotides	phosphoribosylaminoimidazolecarboxamide formyltransferase/IMP cyclohydrolase

TIGR01737	FGAM_synth_I	Purines, pyrimidines, nucleosides, and nucleotides	phosphoribosylformylglycinamidine synthase I
TIGR00485	EF-Tu	Protein synthesis	translation elongation factor Tu
TIGR00635	ruvB	DNA metabolism	Holliday junction DNA helicase RuvB
TIGR01066	rplM_bact	Protein synthesis	ribosomal protein uL13
TIGR01067	rplN_bact	Protein synthesis	ribosomal protein uL14
TIGR00962	atpA	Energy metabolism	ATP synthase F1, alpha subunit
TIGR01280	xseB	DNA metabolism	exodeoxyribonuclease VII, small subunit
TIGR00430	Q_tRNA_tgt	Protein synthesis	tRNA-guanine transglycosylase
TIGR01060	eno	Energy metabolism	phosphopyruvate hydratase
TIGR00967	3a0501s007	Protein fate	preprotein translocase, SecY subunit
TIGR01009	rpsC_bact	Protein synthesis	ribosomal protein uS3
TIGR00670	asp_carb_tr	Purines, pyrimidines, nucleosides, and nucleotides	aspartate carbamoyltransferase
TIGR00082	rbfA	Transcription	ribosome-binding factor A
TIGR00168	infC	Protein synthesis	translation initiation factor IF-3
TIGR00436	era	Protein synthesis	GTP-binding protein Era
TIGR00382	clpX	Protein fate	ATP-dependent Clp protease, ATP-binding subunit ClpX
TIGR00460	fmt	Protein synthesis	methionyl-tRNA formyltransferase
TIGR00064	ftsY	Protein fate	signal recognition particle-docking protein FtsY
TIGR00061	L21	Protein synthesis	ribosomal protein bL21
TIGR00337	PyrG	Purines, pyrimidines, nucleosides, and nucleotides	CTP synthase
TIGR00166	S6	Protein synthesis	ribosomal protein bS6
TIGR00062	L27	Protein synthesis	ribosomal protein bL27
TIGR01394	TypA_BipA	Regulatory functions	GTP-binding protein TypA/BipA
TIGR00416	sms	DNA metabolism	DNA repair protein RadA
TIGR02386	rpoC_TIGR	Transcription	DNA-directed RNA polymerase, beta' subunit
TIGR00755	ksgA	Protein synthesis	ribosomal RNA small subunit methyltransferase A
TIGR00029	S20	Protein synthesis	ribosomal protein bS20
TIGR00212	hemC	Biosynthesis of cofactors, prosthetic groups, and carriers	hydroxymethylbilane synthase
TIGR00435	cysS	Protein synthesis	cysteine--tRNA ligase

TIGR00560	pgsA	Fatty acid and phospholipid metabolism	CDP-diacylglycerol-- glyecrol-3-phosphate 3-phosphatidyltransferase
TIGR03263	guanyl_kin	Purines, pyrimidines, nucleosides, and nucleotides	guanylate kinase
TIGR00302		Purines, pyrimidines, nucleosides, and nucleotides	
TIGR00008	infA	Protein synthesis	translation initiation factor IF-1
TIGR01455	glmM	Central intermediary metabolism	phosphoglucosamine mutase
TIGR00952	S15_bact	Protein synthesis	ribosomal protein uS15
TIGR03594	GTPase_EngA	Protein synthesis	ribosome-associated GTPase EngA
TIGR03632	uS11_bact	Protein synthesis	ribosomal protein uS11
TIGR00362	DnaA	DNA metabolism	chromosomal replication initiator protein DnaA
TIGR00086	smpB	Protein synthesis	SsrA-binding protein
TIGR03654	L6_bact	Protein synthesis	ribosomal protein uL6
TIGR01171	rplB_bact	Protein synthesis	ribosomal protein uL2
TIGR00188	rnpA	Transcription	ribonuclease P protein component
TIGR01173	glmU	Central intermediary metabolism	UDP-N-acetylglucosamine diphosphorylase/glucosamine-1-phosphate N-acetyltranferase
TIGR00580	mfd	DNA metabolism	transcription-repair coupling factor
TIGR01071	rplO_bact	Protein synthesis	ribosomal protein uL15
TIGR01011	rpsB_bact	Protein synthesis	ribosomal protein uS2
TIGR00191	thrB	Amino acid biosynthesis	homoserine kinase
TIGR00595	priA	DNA metabolism	primosomal protein N'
TIGR00690	rpoZ	Transcription	DNA-directed RNA polymerase, omega subunit
TIGR01032	rplT_bact	Protein synthesis	ribosomal protein bL20
TIGR02191	RNaseIII	Transcription	ribonuclease III

Table S3: Genome sequenced quality profile of the *B. vallismortis* E4.

Properties	Tool/ Value
Assembler	SPAdes
Completeness	100.0
Contamination	0.16
Completeness_Model_Used	Neural Network (Specific Model)11
Translation_Table_Used	0.883
Coding_Density	306176
Contig_N50	297.74074074074076

Genome Size	4145336
GC_Content	44%
Total_Contigs	76
Total_Coding_Sequences	4104
Max_Contig_Length	762729
Avg. coverage depth	276
GenBank accession	JBIMFA000000000
Read counts	4.2M
Total bases	1.2G bases
Sequencing depth	~293x

Table S4. Summary of functional annotation of *Bacillus vallismortis* E4 proteome, showing enzymes (EC), gene ontology (GO), metabolic pathway, and PATRIC family assignments.

Hypothetical proteins	715
Proteins with functional assignments	3,508
Proteins with EC number assignments	1,045
Proteins with GO assignments	869
Proteins with Pathway assignments	766
Proteins with PATRIC genus-specific family (PLfam) assignments	3,900
Proteins with PATRIC cross-genus family (PGfam) assignments	3,941

Figure S1. Pie chart illustrating the metabolic classes of annotated genes present in the draft genome of *B. vallismortis* E4.

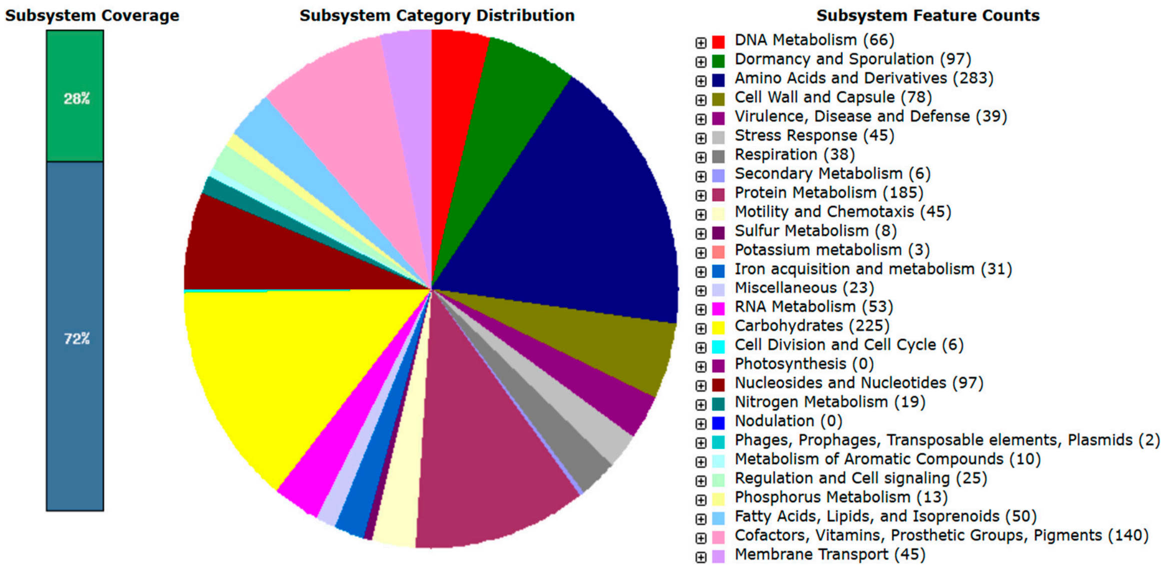

Figure S2: Figure representing the analysis of pathogenicity of *B. vallismortis* E4 on the basis of neural network principle using PathogenFinder2.

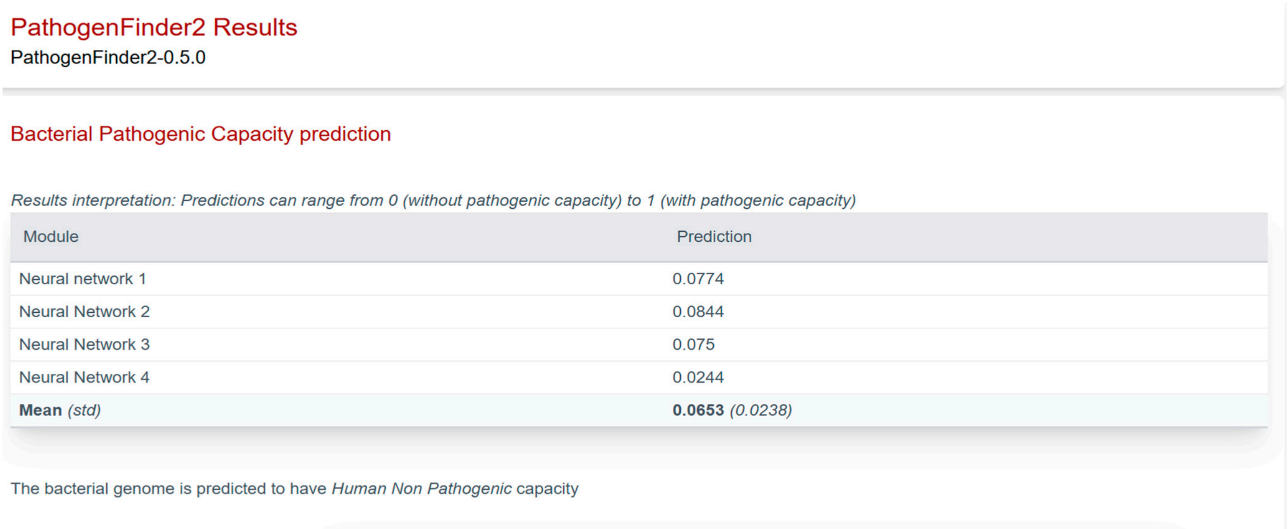

PathogenFinder2 Results

PathogenFinder2-0.5.0

Bacterial Pathogenic Capacity prediction

Results interpretation: Predictions can range from 0 (without pathogenic capacity) to 1 (with pathogenic capacity)

Module	Prediction
Neural network 1	0.0774
Neural Network 2	0.0844
Neural Network 3	0.075
Neural Network 4	0.0244
**Mean** *(std)*	**0.0653** *(0.0238)*

The bacterial genome is predicted to have *Human Non Pathogenic* capacity

Figure S3: Biosurfactant analysis: (A) is exhibiting Oil displacement area in the presence of biosurfactant produced by *B. vallismortis* E4, (B) is showing emulsification of the olive oil in the presence of biosurfactant produced by *B. vallismortis* E4.

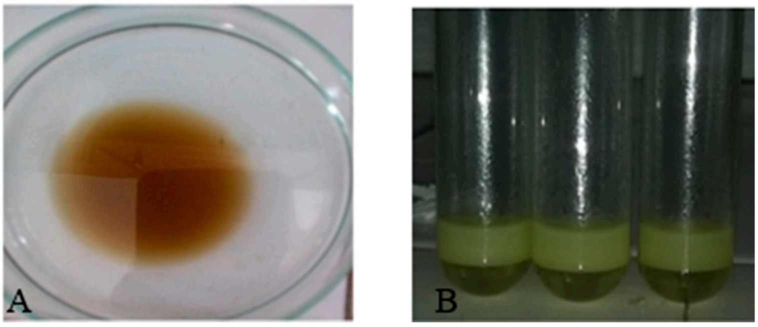
A
B
